# Adaptation of an inpatient CHW-led Care Transition Program for Persons with Dementia and Caregivers

**DOI:** 10.1093/geroni/igaf122.2845

**Published:** 2025-12-31

**Authors:** Jung Kwak, Allison Salter, Aveen Ghodsi Jafari, Andrea Perry, Alexis Shepherd, Snehal Patel, Sarah Stayer

**Affiliations:** University of Texas-Austin, Austin, Texas, United States; The University of Texas at Austin, Austin, Texas, United States; The University of Texas at Austin, Austin, Texas, United States; Raymond G. Murphy Department of Veterans Affairs Medical Center, Albuquerque, New Mexico, United States; Dell Medical School at The University of Texas at Austin, Austin, Texas, United States; Dell Medical School at The University of Texas at Austin, Austin, Texas, United States; Dell Medical School at The University of Texas at Austin, Austin, Texas, United States

## Abstract

Persons with dementia (PWD) and their families frequently face significant challenges during hospital-to-home transitions, highlighting the need for tailored transitional care. This qualitatively driven, nested mixed-methods study applied the Framework for Reporting Adaptations-Enhanced (FRAME) to guide the adaptation of a hospital-based, community health worker (CHW)-led care transition program for hospitalized PWD and their caregivers. The goal was to improve feasibility, acceptability, and implementation fidelity. Adaptations were informed by key informant interviews (n = 5), a community advisory board of hospital providers, social services experts, and caregivers (n = 11), and a caregiver needs assessment (n = 36). Data were analyzed using thematic content analysis and descriptive statistics. Key adaptations included: (1) expanding the community health worker’s (CHW) role to provide caregiver education, emotional support, and personalized coaching; (2) enhancing assessments to cover caregiver concerns, resource needs, ADL/IADL support, and home safety; (3) allowing flexible timing for initial assessments post-discharge; (3) broadening eligibility to include individuals without a formal dementia diagnosis; and (4) developing multilingual recruitment materials (English/Spanish), brief introduction videos, and hospital-based outreach through case management teams to improve participation. This presentation discusses stakeholder engagement, key intervention modifications, recruitment challenges, and practical insights for implementing and scaling CHW-led transitional care for PWD and caregivers.

